# Orthographic Knowledge as a Predictor of Writing Composition in European Portuguese: A Longitudinal Study in Grade 2

**DOI:** 10.3390/bs16050652

**Published:** 2026-04-26

**Authors:** Luís Querido, Sandra Fernandes, Arlette Verhaeghe, Catarina Marques

**Affiliations:** 1 Egas Moniz Center for Interdisciplinary Research (CiiEM), Egas Moniz School of Health & Science, Caparica, 2829-511 Almada, Portugal; 2 Faculdade de Psicologia, Universidade de Lisboa, Alameda da Universidade, 1649-013 Lisboa, Portugal; sfernandes@psicologia.ulisboa.pt (S.F.); averhaeghe@psicologia.ulisboa.pt (A.V.); 3 CICPSI, Faculdade de Psicologia, Universidade de Lisboa, Alameda da Universidade, 1649-013 Lisboa, Portugal; 4 Business Research Unit (BRU-ISCTE), Iscte—Instituto Universitário de Lisboa, 1649-026 Lisboa, Portugal; catarina.marques@iscte-iul.pt

**Keywords:** lexical orthographic knowledge, sublexical orthographic knowledge, spelling, written composition, European Portuguese

## Abstract

Writing development in the early grades depends critically on transcription skills, yet little is known about how components of orthographic knowledge support children’s written composition in European Portuguese. This study examined whether lexical and sublexical orthographic knowledge assessed at the beginning of Grade 2 predict written composition at the end of the school year, and whether these effects are direct or mediated by word spelling. Eighty Grade 2 children completed measures of lexical orthographic knowledge (orthographic choice), sublexical orthographic knowledge (orthographic awareness), and word spelling at the beginning of the year, and a written composition task scored for lexical diversity at year’s end. Path analyses with maximum likelihood estimation and bias-corrected bootstrapping showed that orthographic knowledge explained 44% of the variance in word spelling and up to 18% in written composition. Lexical orthographic knowledge was a significant direct predictor of written composition (*β* = 0.38, *p* < 0.01), whereas sublexical orthographic knowledge showed a small but significant indirect effect through spelling (*β* = 0.08, *p* < 0.05) in the full mediation model. These findings highlight the central role of orthographic knowledge, particularly its lexical component, in supporting early writing in an orthography of intermediate depth.

## 1. Introduction

Writing development in the early grades relies heavily on children’s ability to coordinate transcription processes, handwriting and spelling, with emerging linguistic and cognitive skills (Berninger & Swanson, 1994; Kim, 2020). Developmental models of writing consistently highlight that, during the first years of schooling, transcription processes consume substantial cognitive resources and therefore strongly constrain children’s capacity to generate written text (Berninger et al., 1998; Berninger & Winn, 2006). As these skills gradually become automatized, children can allocate more resources to higher-level writing processes such as planning, revising, and text organization (Fayol, 2012). Within this developmental framework, spelling has been identified as one of the most robust predictors of children’s writing performance, including text length, productivity, and global writing quality (Abbott et al., 2010; Graham et al., 1997). Beyond handwriting, the contribution of spelling to writing has been observed both in typically developing writers and in children with dyslexia (Berninger et al., 2008). Longitudinal evidence shows that spelling remains one of the most stable writing-related skills across childhood (Abbott et al., 2010), supporting the notion that spelling serves as a foundational component of writing development.

In addition to these developmental constraints, several studies have shown that children’s ability to generate written text depends on the efficient coordination of linguistic knowledge and transcription skills, with spelling functioning as a developmental bottleneck for young writers (e.g., McCutchen, 2006). Research consistently demonstrates that writers who allocate fewer cognitive resources to spelling are able to produce longer, more complex, and more coherent texts, reinforcing spelling as a central mechanism through which early writing proficiency emerges (Connelly et al., 2005; Limpo & Alves, 2013). This body of work is consistent with contemporary multicomponential models of writing (e.g., Hayes, 2012; Kim, 2020), which emphasize that transcription processes interact with linguistic, cognitive, and discourse-level resources in dynamic ways that shift across development. In early primary school, these interactions are particularly constrained because children’s handwriting fluency, orthographic processing, and morphological knowledge are still fragile, making the quality of transcription disproportionately influential. More recent theoretical and empirical work has reinforced these claims by showing that the developmental impact of transcription skills on written composition remains particularly pronounced in the early grades, when children’s working-memory resources are still constrained and writing processes are not yet efficiently coordinated (e.g., Kim, 2020). These contemporary perspectives converge in highlighting orthographic learning and fluent access to word forms as key mechanisms enabling children to move from resource-consuming transcription to more generative text production.

From a process perspective, transcription skills constitute lower-level mechanisms that can constrain higher-level composition, particularly during the early stages of writing development (Berninger & Swanson, 1994; McCutchen, 2006). When transcription becomes more automatic, working memory and attentional resources can be reallocated to higher-level processes involved in text generation, including lexical selection, sentence formulation, and discourse organization (Berninger et al., 1998; Fayol, 2012; Hayes, 2012). Therefore, individual differences in orthographic knowledge may constrain writing not only through accuracy outcomes, but also through the efficiency with which writers retrieve and deploy lexical representations during composition, thereby influencing the linguistic richness and quality of written output (Kim, 2020; Kim & Schatschneider, 2017).

Recent evidence further suggests that children’s transcription skills are intimately linked to the efficiency with which they retrieve and manipulate linguistic representations during writing (e.g., Kim & Schatschneider, 2017). In early writing, these mechanisms include the efficiency of accessing stored orthographic word forms, coordinating phonological-to-orthographic mappings, and integrating morpho-orthographic information during lexical retrieval, processes that directly affect how much cognitive capacity remains available for higher-level text generation (Kim & Schatschneider, 2017; Limpo & Alves, 2013; McCutchen, 2006). These findings highlight the need to examine not only spelling accuracy but also the underlying cognitive and linguistic mechanisms that enable children to generate, retrieve, and produce written forms. Among these mechanisms, orthographic knowledge has emerged as one of the most theoretically and empirically promising. During early stages of literacy acquisition, when transcription processes remain highly resource-demanding, individual differences in orthographic efficiency may have an especially pronounced impact on children’s ability to produce written text. Moreover, research on children’s writing has shown that more efficient spelling and orthographic processing are associated with richer lexical use and greater structural complexity in written compositions (Jarvis, 2002; Johansson, 2008; McNamara et al., 2010). Recent studies have further highlighted that orthographic knowledge supports not only the accuracy of individual word spellings but also lexical retrieval speed, morphological integration, and sentence-level choices during text production (Kim & Schatschneider, 2017; Pacton & Commissaire, 2024; González, 2017). Together, these findings suggest that orthographic knowledge may exert broader and more multifaceted influences on writing than those captured by transcription accuracy alone.

### 1.1. Orthographic Knowledge as a Foundational Component of Spelling

Research has consistently shown that orthographic knowledge is a key foundation for spelling development (Conrad et al., 2013; Rothe et al., 2014; Querido et al., 2021), and that spelling, in turn, is one of the strongest predictors of children’s writing productivity and quality (Abbott et al., 2010; Graham et al., 1997). Orthographic knowledge is defined as the set of lexical and sublexical representations that specify how words and letter patterns are legally formed in a writing system (e.g., Apel, 2011; Frith, 1980). It is considered a low-level literacy skill because it operates at the word and sub-word level, supporting accurate and efficient processing of written forms. In contrast, high-level literacy skills (e.g., reading comprehension, written composition) involve meaning construction and discourse organization. However, low-level skills are foundational: efficient orthographic processing frees cognitive resources for higher-level processes (LaBerge & Samuels, 1974; Perfetti, 2007). Orthographic knowledge includes (a) lexical knowledge, which supports recognition and retrieval of word-specific spellings, and (b) sublexical knowledge, which involves sensitivity to orthographic patterns, positional constraints, and permissible letter sequences (e.g., Deacon et al., 2012, 2014; Querido et al., 2020). In line with this dual-component view, tasks that require discrimination among correct spellings and closely related foils are commonly used to index the quality of stored, word-specific orthographic representations (lexical orthographic knowledge). In contrast, tasks that require judging the legality of letter sequences in pronounceable nonwords are used to capture sensitivity to graphotactic/orthotactic regularities and positional constraints that generalize beyond specific words (sublexical orthographic knowledge), which is often assumed to be acquired through exposure to print and statistical learning mechanisms. Both components have been shown to uniquely predict spelling ability across languages, including English (Conrad et al., 2013), German (Rothe et al., 2014; Zarić et al., 2021), and Portuguese (Querido et al., 2021). Evidence from other alphabetic orthographies further suggests that the relative contribution of lexical versus sublexical orthographic knowledge to writing varies as a function of orthographic consistency and morphological demands (e.g., Babayiğit & Stainthorp, 2010; Vaessen et al., 2010). In more transparent systems, sublexical mappings may early support accuracy for spelling novel items, whereas in less consistent or morphologically richer systems, word-specific representations and morpho-orthographic regularities become increasingly critical for both spelling and written production. Importantly, prior longitudinal work (Querido et al., 2021) demonstrated that lexical orthographic knowledge becomes increasingly dominant across grades, whereas sublexical knowledge plays a relatively stronger role only at earlier stages.

The developmental differentiation between lexical and sublexical components has been observed across alphabetic systems of varying orthographic depth. In English, orthographic pattern knowledge contributes significantly to spelling across childhood (Conrad et al., 2013), and in German, early sensitivity to orthographic regularities predicts spelling accuracy at the end of Grade 1 (Rothe et al., 2014). In European Portuguese, longitudinal evidence demonstrates that lexical orthographic knowledge is particularly influential for word-specific spelling, especially from Grade 2 onward, while sublexical orthographic knowledge tends to contribute primarily to pseudoword spelling and early decoding processes (Querido et al., 2021). Together, these findings support the idea that orthographic knowledge is multidimensional and develops in a gradual progression from sublexical pattern sensitivity toward reliance on word-specific lexical representations. Cross-linguistic longitudinal evidence further indicates that the balance between sublexical and lexical orthographic knowledge is shaped by orthographic depth and morphological complexity. In relatively consistent orthographies, sublexical knowledge supports early spelling, but lexical orthographic representations increasingly account for variance in spelling and written production as children progress through primary school (Caravolas et al., 2012; Georgiou et al., 2012). In less consistent or more morphologically constrained orthographies, lexical orthographic knowledge tends to become a stronger predictor earlier, reflecting increased demands for storing word-specific spellings and context-sensitive patterns (Conrad et al., 2013; Treiman & Kessler, 2014).

This perspective is further reinforced by research on statistical learning (e.g., graphotactic regularities), which shows that children implicitly acquire sensitivity to permissible letter patterns through exposure to print (Pacton & Commissaire, 2024). Such implicit learning strengthens sublexical orthographic representations and constrains children’s spelling attempts even before explicit rule knowledge develops. Recent work in cross-linguistic settings (e.g., Querido et al., 2021; Zarić et al., 2021) also demonstrates that the developmental utility of lexical versus sublexical orthographic knowledge varies according to the orthographic transparency. Notably, in orthographies of intermediate depth, such as European Portuguese, children must rely heavily on both types of knowledge, given the coexistence of regular phoneme–grapheme mappings and substantial spelling irregularities (Fernandes et al., 2008; Sucena et al., 2009). In European Portuguese, this intermediate-depth profile is often expressed as an asymmetry between reading and spelling: while many grapheme–phoneme correspondences support relatively efficient decoding, spelling requires selecting among alternative graphemic options and applying positional/contextual constraints, making encoding comparatively more demanding and more dependent on orthographic knowledge (e.g., Querido et al., 2021). This suggests that the study of orthographic knowledge in Portuguese offers an ideal opportunity to examine the distinct pathways through which lexical and sublexical processes support literacy development.

### 1.2. Orthographic Knowledge and Written Composition

Although the influence of orthographic knowledge on spelling is well established, far less is known about whether orthographic knowledge also contributes, directly or indirectly, to children’s written composition. This is particularly relevant in European Portuguese, a language of intermediate orthographic depth where orthographic regularities and irregularities shape early spelling development (e.g., Querido et al., 2021). Theoretically, it is plausible that orthographic knowledge influences writing through two pathways: (a) indirectly, by supporting spelling accuracy, and (b) directly, by facilitating faster access to word forms during text production, thereby reducing the transcription burden.

In addition to its potential contributions to transcription efficiency, orthographic knowledge may also influence the linguistic quality of children’s written output.

Research on writing evaluation has increasingly emphasized lexical diversity as a reliable indicator of writing quality, particularly in developing writers (Beard, 1984; Jarvis, 2002; Johansson, 2008). Studies using measures such as Type–Token ratios, D, MATTR, or other robust indices of lexical diversity show that more skilled writers produce texts with a wider range of vocabulary, reflecting more efficient lexical retrieval and richer linguistic representations (McNamara et al., 2010; Olinghouse & Wilson, 2013). Importantly, lexical diversity is strongly associated with vocabulary knowledge, orthographic fluency, and with children’s ability to efficiently access and retrieve specific lexical items during composition, all of which are supported by the quality of lexical orthographic representations (González, 2017; Kim & Schatschneider, 2017; McNamara et al., 2010; Olinghouse & Wilson, 2013). Thus, in languages such as Portuguese, where written word forms vary considerably in transparency, orthographic knowledge may support not only spelling accuracy but also children’s capacity to generate lexically richer texts.

Recent studies further suggest that orthographic knowledge may influence written composition through morphological and lexical pathways. Morphological awareness interacts closely with orthographic learning (Deacon et al., 2014; Kim, 2020), and children with stronger orthographic representations tend to deploy more morphologically complex words in written text (González, 2017). Moreover, research on children’s writing in diverse orthographies indicates that orthographic fluency supports not only productivity but also syntactic complexity, discourse cohesion, and genre-appropriate lexical selection (e.g., Kim, 2020; Limpo & Alves, 2013). These findings suggest that orthographic knowledge may function as a foundational enabler of multiple linguistic processes involved in writing, particularly during early development when cognitive resources are limited.

### 1.3. The Present Study

The present study examined the direct and mediated contributions of lexical and sublexical orthographic knowledge to children’s written composition. We employed a longitudinal design within Grade 2, a developmentally critical period, assessing orthographic knowledge and spelling at the beginning of the school year and written composition at the end. This design allows examination of predictive relationships while controlling for temporal precedence, strengthening predictive inference relative to cross-sectional designs (Little, 2013). We assessed lexical orthographic knowledge with an orthographic choice task requiring discrimination among orthographically similar alternatives, and sublexical orthographic knowledge with an orthographic awareness task requiring judgments about legal versus illegal orthographic patterns in pseudowords. These tasks were selected based on evidence that they tap distinct components of orthographic knowledge (Apel et al., 2012; Cassar & Treiman, 1997) and predict literacy outcomes independently (Conrad et al., 2013).

Lexical diversity, operationalized as the number of different correctly spelled word types, was selected as the writing outcome for three reasons. First, it captures both transcription accuracy and lexical access, reflecting the integration of orthographic and semantic knowledge during text generation (Olinghouse & Wilson, 2013). Second, it is developmentally appropriate for Grade 2, when children transition from transcription-focused to meaning-focused composition (Berninger & Swanson, 1994). Third, it provides a quantifiable index sensitive to individual differences in early writing development (Kim et al., 2013). Although lexical diversity does not capture all dimensions of writing quality (e.g., syntactic complexity, discourse organization), it represents a foundational component reflecting children’s ability to retrieve and accurately transcribe varied vocabulary during text generation.

Spelling was selected as the mediator between orthographic knowledge and written composition for theoretical and empirical reasons. Theoretically, spelling represents the transcription component that translates orthographic representations into written output, making it a plausible mechanism linking orthographic knowledge to text generation (Berninger & Swanson, 1994). Empirically, spelling consistently predicts writing outcomes across development (Abbott et al., 2010; Graham et al., 1997). Although other skills (e.g., handwriting fluency, morphological awareness) also contribute to writing, spelling was prioritized because transcription accuracy is a primary constraint on early writing quality (Graham et al., 1997). Future research should examine whether handwriting fluency and morphological awareness provide additional mediating pathways.

The present study addressed two research questions (RQ):

RQ1. Do lexical and sublexical orthographic knowledge assessed at the beginning of Grade 2 predict children’s written composition at the end of the school year?

RQ2. Are the effects of lexical and sublexical orthographic knowledge on written composition direct, or are they mediated by word spelling?

Based on prior evidence, we formulated two hypotheses:

**H1.**  *If orthographic knowledge supports spelling, and spelling supports writing, it follows that orthographic knowledge may influence written composition both directly and indirectly through its impact on spelling (H1).*

Such dual-pathway conceptualization is consistent with contemporary hierarchical models of writing (e.g., Kim, 2020), which posit that lower-level processes contribute both indirectly through their support for higher-level execution and directly by modulating the efficiency with which linguistic representations are retrieved and deployed. Longitudinal evidence in European Portuguese indicates that lexical orthographic knowledge exerts stronger and more consistent effects on spelling than sublexical knowledge across primary school (Querido et al., 2021). This pattern reflects a developmental shift from reliance on sublexical orthotactic regularities toward increasingly robust lexical representations, which are more directly implicated in written word production. By the end of Grade 2, children typically rely more on stored lexical representations than on sublexical patterns to support accurate spelling and fluent text generation.

**H2.**  *Given this developmental asymmetry and the increasing dominance of lexical representations as children progress through the grades, it is expected that lexical orthographic knowledge will exert a stronger influence on written composition than sublexical orthographic knowledge.*

Hypothesis 2 is also consistent with evidence from other orthographies, such as German (e.g., Zarić et al., 2021) and isiXhosa (Daries & Bowles, 2024), where lexical orthographic knowledge emerges as the most reliable predictor of spelling accuracy by the elementary grades.

**Figure 1:**
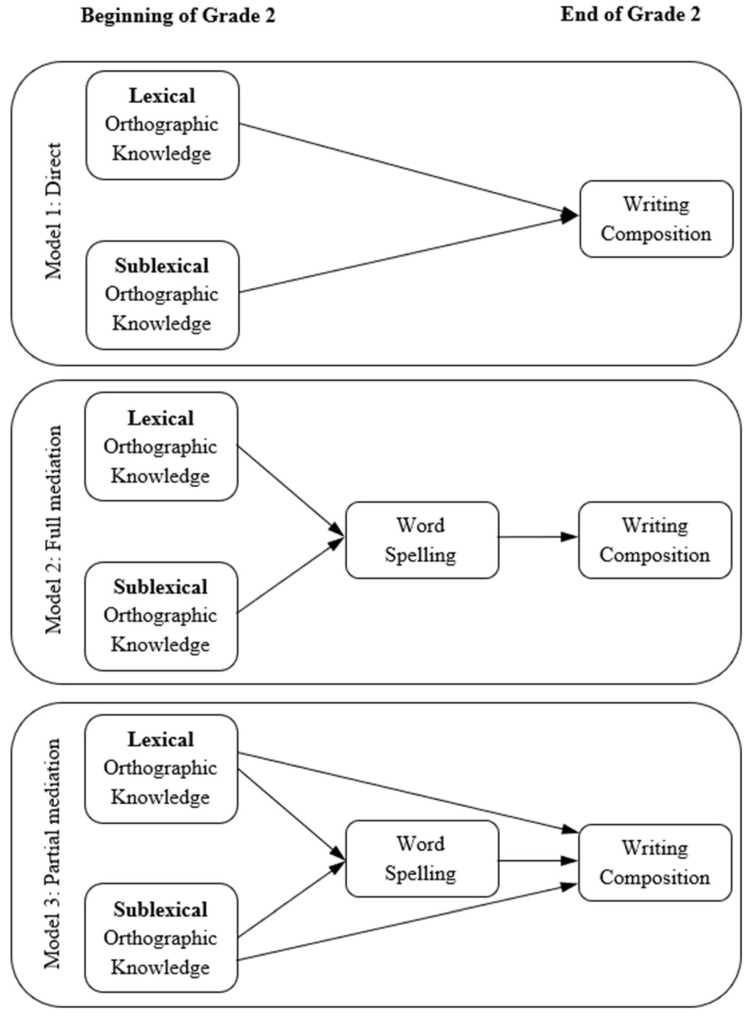
Conceptual models tested in the path analyses. Model 1 specifies direct effects of lexical and sublexical orthographic knowledge on written composition. Model 2 tests a fully mediated model in which the effects of orthographic knowledge on writing occur exclusively through word spelling. Model 3 evaluates a partially mediated model including both direct and indirect paths between orthographic knowledge and written composition. All independent variables were measured at the beginning of Grade 2, and the dependent variable was measured at the end of Grade 2.

To test these hypotheses, we contrasted alternative path models (Figure 1) representing direct, fully mediated, and partially mediated relations between orthographic knowledge and written composition, with all variables measured at the beginning of Grade 2 predicting writing outcomes obtained at the end of Grade 2. This study extends previous research by examining these relations within a single school year, a period in which transcription skills undergo substantial growth, and by addressing a gap in the literature on early writing development in European Portuguese. Focusing on this early developmental phase allows us to capture the point at which orthographic knowledge is theoretically most influential for writing performance, enabling us to assess its direct and mediated effects. Furthermore, by operationalising written composition through lexical diversity, a measure increasingly recognised as a robust indicator of written quality in children (González, 2017; Olinghouse & Wilson, 2013), the present study provides novel evidence regarding how the foundations of orthographic processing shape the lexical expressiveness of children’s written texts. Such an approach offers an important contribution to current debates about how early writing should be evaluated and about which linguistic and cognitive processes best explain individual differences in emergent compositional skill.

## 2. Materials and Methods

### 2.1. Participants

Data for the present study come from a larger project on reading, spelling, and writing development in European Portuguese. The Ministry of Education of the Portuguese Government granted access to the public schools that participated in this project.

A total of 83 children (37 girls, 46 boys) from two public schools in the Lisbon district were initially recruited. Within each school, participants were drawn from two Grade 2 classrooms (i.e., four classrooms in total). The participating schools were located within the same demographic region and implemented the same national curriculum and comparable literacy instruction. All children were enrolled in Grade 2 at the start of the study, and the same cohort was followed longitudinally. Assessments were conducted at two time points: (i) within the first three months of Grade 2; and (ii) within the last three months of Grade 2. These two assessment points examine whether orthographic knowledge measured at the beginning of Grade 2 predicts writing performance at the end of Grade 2.

All participants were native speakers of European Portuguese and exhibited average or above-average nonverbal cognitive functioning, operationalized as scoring at or above the 25th percentile on the Raven’s Colored Progressive Matrices (Simões, 1994). Children flagged by teachers or parents as presenting learning, emotional, behavioural, or sensory difficulties were excluded. These screening procedures resulted in the ineligibility of 15 children from the original recruitment pool.

Of the 83 eligible children, 80 (96.4%) had complete data and were therefore retained for the analyses reported in the present study. Children’s mean age at the end of Grade 2 was 96.45 months (SD = 3.30). Based on parent questionnaires, participating families were generally from middle to upper-middle socioeconomic backgrounds. Instruction in reading and spelling followed a phonics-based approach, the method officially adopted by the participating schools.

All procedures adhered to APA ethical guidelines and were approved by the relevant national and institutional bodies overseeing research with human participants. Parents or legal guardians provided written informed consent consistent with the Office of Statistics and Planning of Education of the Ministry of Education, and children gave verbal assent prior to participation. All assessments were administered individually or in small groups during regular school hours in quiet rooms provided by the schools, following standardized administration procedures.

### 2.2. Measures

#### 2.2.1. Raven’s Colored Progressive Matrices (RCPM)

General nonverbal cognitive ability was assessed using the Portuguese adaptation of the Raven’s Colored Progressive Matrices (Simões, 1994). This instrument provides a robust estimate of fluid reasoning and nonverbal problem-solving ability and is widely used in developmental research with children. The Portuguese adaptation demonstrates excellent internal consistency, with a reported Cronbach’s alpha of 0.91, indicating high reliability.

#### 2.2.2. Word Spelling Task

Word spelling (Querido et al., 2021) was assessed through a dictation task composed of 36 words, divided into three categories: simple regular words (n = 12), complex regular words (n = 12), and irregular words (n = 12). Within each category, six high-frequency words and six low-frequency words were selected using Portulex, a lexical database for European Portuguese (Teixeira & Castro, 2006). High- and low-frequency items were approximately matched for length, containing predominantly disyllabic and trisyllabic words (high frequency: six disyllabic and twelve trisyllabic; low frequency: five disyllabic and thirteen trisyllabic).

Regular words followed the conventional grapheme–phoneme and phoneme–grapheme correspondences of European Portuguese. Irregular words deviated from these regularities either because they contained a grapheme with an atypical phonetic realization (e.g., <s> pronounced /z/ in *trânsito*), or because they included a silent grapheme in non-terminal position (e.g., <h> in *hora*), following criteria used by Sprenger-Charolles et al. (1998) and Fernandes et al. (2008).

Simple words contained only single-letter graphemes (e.g., *fita*). Complex words included at least one multiletter grapheme, specifically a set of digraphs common in European Portuguese: ou, nh, lh, ch, and rr. These digraphs were chosen because they either have possible, although less frequent, alternative spellings (e.g., ou, ch) or because they have no alternative spelling (e.g., nh, lh). These digraphs do not introduce additional difficulty beyond their phoneme–grapheme structure in Portuguese, but they permit the assessment of children’s sensitivity to multiletter orthographic units.

Before the test began, children completed three familiarization items, which were repeated if necessary to ensure understanding of instructions. Each target word was first presented orally within a sentence context to avoid confusion with homophones, after which it was dictated in isolation for transcription.

The internal consistency of the spelling task was high, with a Cronbach’s alpha of 0.86.

#### 2.2.3. Orthographic Choice Task

In the Orthographic Choice Task (Querido et al., 2020), children were presented with 32 sets of five orthographic alternatives displayed vertically on A5 sheets. For each set, they listened to a target word (e.g., *braço*) and were required to select the written form that correctly matched the spoken stimulus. Each set contained the correctly spelled word, a pseudo-homophone foil (e.g., *brasso*), an item that shared visual similarity in the initial syllable (e.g., *draço*), another exhibiting visual similarity in the final syllable (e.g., *braco*), and a nonword formed through an orthotactically illegal combination of the target’s letters (e.g., *arbço*). This configuration allowed for the assessment of children’s ability to discriminate among visually and phonologically similar alternatives, thereby indexing the quality of their lexical orthographic representations. Conceptually, this recognition/discrimination format targets access to stored word-specific spellings under high visual/phonological competition, and is therefore intended to capture lexical orthographic knowledge rather than production-based spelling per se. This task is a recognition/discrimination measure and does not require written production. Thus, it captures the integrity of stored word-specific orthographic representations (lexical orthographic knowledge) rather than children’s ability to generate words in extended text. The internal consistency of the task was high, with Cronbach’s alpha of 0.85.

#### 2.2.4. Orthographic Awareness Task

The Orthographic Awareness Task (Querido et al., 2020) assessed children’s sensitivity to the orthotactic constraints of European Portuguese. Children were presented with 14 pairs of pseudowords (e.g., *prilo–rilop*; *drulo–srulo*), each pair consisting of one item containing a legal orthographic sequence in either initial or final position and another containing a sequence that does not occur in Portuguese. Children were asked to choose the item that “could be a word” or “looks like a word.” Illegal sequences appeared either at the beginning of the pseudoword, as in *srulo* where the onset <sr>—is not permissible, or at the end, as in *rilop*, where <p> cannot occur word-finally in Portuguese. This format targets children’s sensitivity to orthotactic/graphotactic constraints (i.e., whether a letter sequence is permissible in the writing system and in a given position), which is a core component of sublexical orthographic knowledge. This task thus provides a measure of sublexical orthographic knowledge, reflecting children’s implicit awareness of positional constraints on letter combinations. Because items are pronounceable pseudowords, performance relies on sensitivity to the statistical regularities of the writing system (e.g., permissible onsets/codas) rather than on retrieval of specific word spellings, which is precisely the distinction between sublexical pattern knowledge and lexical orthographic representations. Internal consistency for this measure was acceptable, with a Cronbach’s alpha of 0.74.

#### 2.2.5. Written Composition Task

In the Written Composition task (Querido, 2016), children were asked to produce a short composition on a familiar topic (e.g., summer holidays) and were given 30 min to complete the task. Students who finished early were allowed to stop and were not encouraged to extend their texts. This task was designed to resemble curriculum-based writing assessments commonly used in Portuguese primary education.

Written compositions were scored in terms of lexical diversity, operationalized as the number of different words spelled correctly in each child’s text. This operationalization was selected because lexical diversity has been widely used as a linguistically grounded indicator of writing quality in developing writers and can be computed objectively and reliably from children’s texts. Importantly, it is sensitive to lexical selection demands during text production (i.e., the ability to recruit a broader range of word types), which is theoretically relevant when examining how orthographic knowledge may facilitate efficient access to lexical representations during written composition. Although this outcome includes an accuracy criterion at the word level, it derives from a self-generated composition and primarily indexes lexical breadth and lexical selection during text production (i.e., the ability to recruit a wider range of word types). To compute this score, children’s compositions were first transcribed verbatim, preserving original spelling. Lexical diversity was then estimated using the Hermetic Word Frequency Counter (Hermetic Systems, n.d.), a software tool that automatically counts word types and identifies unique correctly spelled forms. This procedure provided an objective and reproducible measure of lexical diversity, minimizing scorer subjectivity and ensuring consistency across compositions.

### 2.3. General Procedure

All assessments were administered in the participating schools during regular instructional hours and were conducted by trained graduate students following standardized administration protocols. Testing was organized into two separate periods within Grade 2, ensuring that each task was administered under conditions that minimized fatigue and maximized comprehension.

At the beginning of Grade 2, children completed the nonverbal cognitive measure (Raven’s Colored Progressive Matrices) as well as the two orthographic knowledge tasks, the Orthographic Choice Task and the Orthographic Awareness Task, and the Word Spelling Task. These assessments were conducted in small groups in quiet rooms provided by the schools. Instructions were delivered collectively, using a scripted protocol, and practice items were presented when necessary to ensure that all children understood the response format before proceeding to the test items.

At the end of Grade 2, children completed the Written Composition Task. This assessment was administered in classroom groups, with the classroom teacher present for behavioural support while the trained graduate examiners conducted the session. The task was delivered following the same script-based instruction procedures to ensure consistency across classrooms.

Testing across classrooms was synchronized to minimize variation in instructional exposure between groups, and data collection in each testing period was completed within a two- to three-week window.

### 2.4. Statistical Analysis: Path Analysis

Path analysis was used to examine the hypothesized relations among lexical orthographic knowledge, sublexical orthographic knowledge, word spelling, and written composition. This analytic approach is particularly appropriate for testing theoretically specified models involving direct and mediated effects among multiple observed variables, allowing estimation of unique contributions while accounting for the shared variance inherent in correlated predictors (Pedhazur, 1997).

Analyses were conducted using AMOS 19.0 (Arbuckle, 2010), with parameters estimated through the maximum likelihood method. To obtain robust estimates of indirect effects and their confidence intervals, we employed the percentile and bias-corrected bootstrap procedures recommended by Preacher and Hayes (2008), generating 2000 bootstrap samples. Prior to estimating the models, multivariate outliers were identified using the squared Mahalanobis distance, and assumptions of normality were evaluated through indices of skewness and univariate and multivariate kurtosis. All variables demonstrated levels of normality acceptable for maximum likelihood estimation. Accuracy variables are reported as proportions to standardize performance across tasks with different numbers of items and response structures. Path analysis allows the specification of structural models that reflect theoretically grounded hypotheses about how variables relate to one another through direct or indirect pathways. In this context, the strength of each relation is represented by a standardized path coefficient, which estimates the amount of unique variance in an outcome variable accounted for by a predictor. Because path analysis incorporates measurement reliability in the estimation process, it provides a more precise assessment of structural relations than analyses based solely on observed correlations.

Bootstrapping, as described by Efron and Tibshirani (1993), enables empirical approximation of the sampling distribution of indirect effects by repeatedly resampling from the observed dataset. The percentile and bias-corrected methods used here yield confidence intervals for indirect effects that do not assume normality, enhancing the accuracy of mediation testing and supporting strong inferences regarding specific and total indirect effects (Preacher & Hayes, 2008).

The three nested models depicted in Figure 1 were estimated to evaluate the structure of relations within Grade 2. In Model 1, lexical and sublexical orthographic knowledge were specified as direct predictors of written composition. Model 2 tested a full mediation model, in which both components of orthographic knowledge were hypothesized to influence written composition only indirectly, through their effects on word spelling. Model 3, the partial mediation model, assessed whether word spelling mediated the effect of orthographic knowledge while allowing for simultaneous direct paths from lexical and sublexical orthographic knowledge to written composition.

Evidence for mediation was evaluated by comparing the magnitude of the total effect in Model 1 (path c) with the direct effect in Model 3 (path c′). A reduction of c′ relative to c indicates mediation; complete mediation occurs when c′ = 0, whereas partial mediation is indicated when c′ remains significantly different from zero but is smaller than the total effect. Consistent with this logic, Model 1 and Model 2 were estimated primarily for interpretive and statistical comparison, with Model 3 providing the most comprehensive test of the theoretically expected structure of relations.

Significance testing of each path coefficient was performed using the *t* statistic generated by AMOS. Confidence intervals for indirect effects were obtained through the bootstrap procedures described above.

## 3. Results

### 3.1. Descriptive Statistics and Correlational Analyses

Descriptive statistics for all measures administered at the beginning and end of Grade 2 are presented in Table 1. For the Orthographic Choice Task and the Orthographic Awareness Task, accuracy corresponds to the proportion of correct responses, and chance-corrected accuracy scores are additionally reported to account for the probability of correct guessing given the structure of each task (0.20 and 0.50, respectively). Mean performance on the Word Spelling Task and lexical diversity scores for the Written Composition Task (expressed as the total number of different correctly spelled word types) are also included.

Table 2 displays the correlation matrix for all variables considered in the analyses. As expected, moderate associations emerged among the orthographic measures, and both orthographic knowledge and word spelling showed positive correlations with the measure of written composition. All correlations reported in Table 2 were computed using the sample of children with complete data (*N* = 80).

### 3.2. Path Model Results: Relations Among Orthographic Knowledge, Spelling, and Written Composition

**Figure 2:**
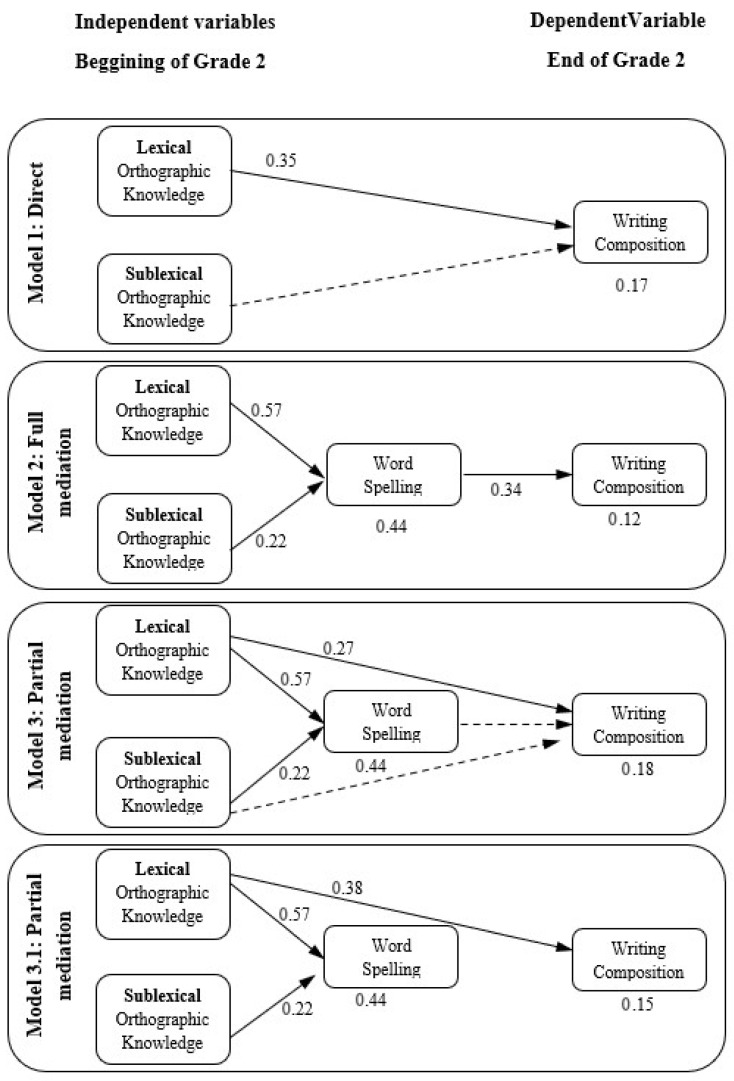
Path diagram of proposed models depicting relationships with significant path coefficient estimates. Solid arrows represent significant paths, whereas dashed arrows represent non-significant paths tested in the models.

The hypothesized structural models were estimated to examine the contribution of lexical and sublexical orthographic knowledge, assessed at the beginning of Grade 2, to children’s written composition at the end of Grade 2. To address H1, we compared models specifying direct paths from orthographic knowledge to writing with models in which effects are mediated by word spelling. To address H2, we evaluated whether lexical orthographic knowledge shows stronger predictive effects on writing outcomes than sublexical orthographic knowledge across these alternative model specifications. Figure 2 displays the path diagrams for the three proposed models and the trimmed model (Model 3.1). In the diagrams, non-significant paths are represented with dashed lines, standardized path coefficients (*β*) are shown adjacent to each arrow, and the squared multiple correlations (*R*^2^) of endogenous variables appear beneath each corresponding box.

All variables exhibited acceptable levels of univariate and multivariate normality based on skewness and kurtosis statistics. A small number of multivariate outliers were identified using the squared Mahalanobis distance and removed prior to model estimation. Analyses were conducted using maximum likelihood estimation in AMOS 19.0, and indirect effects were evaluated using percentile and bias-corrected bootstrapping (2000 samples), following recommendations by Preacher and Hayes (2008).

#### 3.2.1. Model Fit

Table 3 presents the fit indices for the three structural models and the trimmed partial mediation model. All estimated models showed good fit to the data. Chi-square values ranged from 0.00 to 5.34 and were non-significant for all models, indicating that the proposed structures did not significantly deviate from the observed covariance matrix. The Comparative Fit Index (CFI) was excellent across models (≥0.95), and RMSEA values were within the range generally interpreted as acceptable to good fit.

Although Models 1 and 3 yielded saturated solutions (χ^2^ = 0), Model 3.1 exhibited the best combination of statistical adequacy and parsimony, with χ^2^ (2) = 2.61, *p* = 0.27, CFI = 0.99, and RMSEA = 0.06, 90% CFI [0.00, 0.24]. Because Model 3.1 removes only paths unsupported by empirical evidence while retaining the theoretically meaningful structure, it represents the most coherent and best-fitting model describing the relationships among variables within Grade 2. Importantly, the absence of a retained direct path from word spelling to written composition in Model 3.1 should not be interpreted as evidence that spelling is unimportant for writing.

#### 3.2.2. Explained Variance

Across all estimated models, lexical and sublexical orthographic knowledge explained 44% of the variance in word spelling (*R*^2^ = 0.44, *p* < 0.01; see Table 4).

The proportion of variance explained in written composition varied moderately across models (Table 4). When only direct effects were specified (Model 1), lexical and sublexical orthographic knowledge together accounted for 17% of the variance in children’s writing performance. In the full mediation structure (Model 2), the proportion of explained variance was slightly lower (12%), reflecting the more constrained architecture of the model. The partial mediation model (Model 3) yielded the highest explained variance (18%), whereas the simplified partial mediation model (Model 3.1), which includes only statistically meaningful paths, accounted for 15% of the variance in written composition.

#### 3.2.3. Direct and Indirect Effects

Table 5 presents the standardized path coefficients and the corresponding 90% bias-corrected bootstrapped confidence intervals for all estimated models. The results for lexical and sublexical orthographic knowledge are summarised below in textual form to facilitate interpretation.

In accordance with H1, lexical orthographic knowledge displayed, across all models, consistent associations with written composition. In the direct-effects model, the path from lexical orthographic knowledge to written composition was statistically significant (*β* = 0.35, *p* < 0.01). In the full mediation model, the indirect pathway through word spelling was significant (*β* = 0.20, 90% CI [0.10, 0.30], *p* < 0.01). In the partial mediation model, the direct effect was estimated at *β* = 0.27 (90% CI [0.05, 0.49], *p* = 0.06), while the indirect effect through word spelling was not statistically significant (*β* = 0.07, 90% CI [−0.04, 0.19]). The total effect for this model was *β* = 0.35 (90% CI [0.15, 0.52], *p* < 0.01). In the trimmed partial mediation model, which retains only significant pathways, the direct path from lexical orthographic knowledge to written composition was significant (*β* = 0.38, 90% CI [0.20, 0.53], *p* < 0.01).

Sublexical orthographic knowledge did not show significant direct effects on written composition. In the direct-effects model, the estimated path was *β* = 0.14 (n.s.). In the partial mediation model, the estimated direct effect was *β* = 0.11 (n.s.). However, in the full mediation model, the indirect path from sublexical orthographic knowledge to written composition through word spelling was statistically significant (*β* = 0.08, 90% CI [0.02, 0.15], *p* < 0.05).

Across the structural models estimated, lexical and sublexical orthographic knowledge were found to contribute to writing-related outcomes through distinct pathways. Lexical orthographic knowledge demonstrated statistically significant direct associations with written composition across models. Word spelling did not constitute a significant mediator of the relation between lexical orthographic knowledge and writing. Sublexical orthographic knowledge showed no direct relation with written composition in any of the estimated models. However, a statistically significant indirect effect through word spelling was observed in the full mediation model. Orthographic knowledge collectively accounted for 44% of the variance in word spelling. Among the models evaluated, the trimmed partial mediation model (Model 3.1) provided the most parsimonious structure while retaining statistically supported paths.

## 4. Discussion

The present study examined whether children’s written composition at the end of Grade 2 is supported by earlier orthographic knowledge, and whether this influence is exerted directly or through word spelling. Focusing on a single, developmentally critical interval, from the beginning to the end of Grade 2, allowed us to test these relations at a stage when transcription processes are still highly resource-demanding and are expected to play a central role in writing development (Berninger & Winn, 2006; Fayol, 2012).

Consistent with our first hypothesis, the results showed that orthographic knowledge contributes to individual differences in children’s written composition, operationalized as lexical diversity. The final trimmed model indicated that lexical and sublexical orthographic knowledge assessed at the beginning of Grade 2 together explained 44% of the variance in word spelling, and that these predictors accounted for 15% of the variance in written composition at the end of Grade 2. These values are consistent with the idea that, in the early grades, transcription-related skills constitute an important constraint on children’s ability to produce written text (Abbott et al., 2010; Berninger et al., 1998).

These findings are also coherent with contemporary multicomponential models of writing (e.g., Kim, 2020), which emphasize that children’s ability to generate text during the early grades depends on the efficiency with which they retrieve and coordinate linguistic representations at multiple levels, i.e., lexical, orthographic, morphological, and syntactic. In this sense, our results provide additional empirical support for the idea that orthographic processing functions as a bottleneck mechanism constraining the emergence of higher-level writing processes during Grade 2, particularly under conditions of limited cognitive resources.

### 4.1. Orthographic Knowledge, Spelling, and Written Composition

A central finding of this study concerns the role of lexical orthographic knowledge. In the direct-effects model, lexical orthographic knowledge significantly predicted written composition (*β* = 0.35, *p* < 0.01), and in the trimmed model this direct effect was even stronger (*β* = 0.38, *p* < 0.01). These results indicate that children who possess more robust word-specific orthographic representations at the beginning of Grade 2 tend to produce written texts with a greater number of different correctly spelled word types at the end of the school year. This pattern is compatible with theoretical accounts that emphasize the role of orthographic representations in freeing processing resources for written language production and enabling more efficient lexical retrieval during composition (Connelly et al., 2006, 2012).

In contrast to what would be expected under a strictly mediated model, word spelling did not function as a significant mediator of the relation between lexical orthographic knowledge and written composition. In the full mediation model, the indirect pathway from lexical orthographic knowledge to written composition via word spelling was statistically significant (*β* = 0.20, *p* < 0.01), but when both direct and indirect paths were included (partial mediation model), the indirect effect was no longer significant (*β* = 0.07, n.s.). The total effect remained essentially unchanged (*β* = 0.35, *p* < 0.01). This pattern suggests that the main contribution of lexical orthographic knowledge to writing, at least in this developmental window and with lexical diversity as outcome, is direct rather than mediated by spelling performance. This direct influence is consistent with recent evidence indicating that lexical orthographic representations contribute not only to spelling accuracy but also to rapid lexical retrieval, morphological integration, and the selection of more sophisticated lexical items during composition (e.g., González, 2017; Kim & Schatschneider, 2017). These findings converge with the view that lexical-level orthographic processing underpins multiple linguistic subcomponents of writing, thereby exerting a broader effect on written output than that captured by spelling alone.

Sublexical orthographic knowledge showed a different profile. It did not directly predict written composition in any of the models tested (e.g., *β* = 0.14 and *β* = 0.11 for the direct effects in Models 1 and 3, respectively, both n.s.). However, in the full mediation model, sublexical orthographic knowledge had a small but significant indirect effect on written composition via word spelling (*β* = 0.08, *p* < 0.05). This finding is consistent with the notion that sensitivity to legal and illegal letter patterns primarily supports spelling accuracy, which can then have downstream consequences for written text production (Conrad et al., 2013; Deacon et al., 2012, 2014). Moreover, the fact that sublexical orthographic knowledge contributed exclusively through mediation aligns with recent work on statistical learning and graphotactic regularities (Pacton & Commissaire, 2024), which shows that sublexical pattern knowledge provides early scaffolding for spelling but becomes less central once lexical-specific orthographic representations consolidate. Thus, the functional role of sublexical knowledge in writing may be developmentally restricted, supporting spelling during early phases but exerting limited influence on children’s compositional abilities by the end of Grade 2.

Taken together, these results support a differentiated view of orthographic knowledge in writing. Lexical orthographic knowledge appears to be closely tied to written composition, contributing directly to the lexical diversity of children’s texts. In contrast, sublexical orthographic knowledge contributes more modestly and indirectly through spelling. This pattern is in accordance with broader developmental frameworks of literacy, which posit that as children accumulate word-specific orthographic representations, these representations increasingly support complex literacy tasks, including both reading and writing (e.g., Connelly et al., 2012; Ehri, 1997; Share, 1995).

### 4.2. Lexical Versus Sublexical Orthographic Knowledge

Our second hypothesis predicted that lexical orthographic knowledge would exert a stronger influence on written composition than sublexical orthographic knowledge. The present findings are consistent with this prediction. Lexical orthographic knowledge showed substantial associations both with spelling (*β* = 0.57, *p* < 0.01) and with written composition, whereas the contribution of sublexical orthographic knowledge to spelling was smaller (*β* = 0.22, *p* < 0.01) and its relation to writing was exclusively indirect and weak.

The asymmetry between lexical and sublexical components may reflect a shift in the functional role of orthographic knowledge across grades. Early in literacy acquisition, sublexical pattern knowledge supports the decoding and spelling of novel items by constraining possible letter combinations (e.g., Apel, 2011). Over time, however, repeated exposure to print and phonological recoding episodes promotes the consolidation of word-specific orthographic representations (Share, 1995, 2004), which then become the primary basis for both fluent word recognition and accurate spelling. The present study suggests that this same lexically based orthographic system also supports writing, presumably by allowing writers to retrieve word forms efficiently and to deploy a broader range of lexical items during composition.

Concerning spelling, previous longitudinal studies in European Portuguese have also reported that lexical orthographic knowledge becomes particularly important for the development of word-specific spelling from Grade 2 onward, whereas sublexical orthographic knowledge plays a more circumscribed role, especially for pseudoword spelling and earlier phases of decoding (Querido et al., 2021). Complementarily, Querido et al. (2020) study reported robust reciprocal relations between lexical and sublexical orthographic components, suggesting that although both systems interact dynamically during early literacy acquisition, lexical orthographic knowledge becomes increasingly central as children progress through the earlier grades. Taken together, this set of evidence supports the view that in an intermediate depth orthography, literacy development is characterized by a gradual change from reliance on sublexical orthographic knowledge toward more stable, word-specific orthographic representations.

Importantly, the present results extend this developmental pattern from reading and spelling to writing, indicating that lexical orthographic knowledge also underpins children’s ability to generate lexically diverse written texts. This interpretation is consistent with cross-linguistic evidence from alphabetic orthographies with varying degrees of transparency, suggesting that lexical orthographic knowledge increasingly supports writing-related outcomes (e.g., productivity and lexical sophistication) once children go beyond initial decoding and rely more on stored word forms for fluent text production (Berninger et al., 2008; Kim, 2020). In these studies, sublexical orthographic knowledge is typically more strongly linked to spelling accuracy, particularly for unfamiliar strings, whereas lexical orthographic knowledge shows more consistent relations with composing outcomes that depend on efficient lexical access. Similar developmental asymmetries have been documented across other alphabetic systems of varying depth, including German, and isiXhosa (Daries & Bowles, 2024; Ise et al., 2014). Across these languages, lexical orthographic knowledge emerges as the most reliable predictor of writing productivity and lexical sophistication from mid-primary school onward. This cross-linguistic convergence highlights the interpretation that lexical orthographic representations constitute a core foundation for written language generation in alphabetic systems, regardless of specific orthographic transparency.

### 4.3. Orthographic Knowledge, Lexical Diversity, and Writing Quality

An important feature of the present study is the use of lexical diversity, i.e., the number of different correctly spelled word types, as the outcome measure of written composition. Previous research has identified lexical diversity as a core indicator of writing quality in developing writers, often correlating with holistic ratings of organization, coherence, and global quality (Beard, 1984; Jarvis, 2002; Johansson, 2008). Studies using robust diversity indices such as D, MATTR, or similar measures have shown that more skilled writers tend to produce texts with a broader repertoire of lexical items and more varied vocabulary, reflecting richer linguistic representations and more efficient lexical access during writing (McNamara et al., 2010; Olinghouse & Wilson, 2013).

The present findings show that orthographic knowledge assessed early in Grade 2 is associated with this lexical aspect of writing at the end of the school year. This is consistent with the idea, outlined in the introduction, that orthographic knowledge may influence writing not only through accuracy of transcription, but also by facilitating access to a wider range of lexical items during text generation. In European Portuguese, where spelling is more complex than reading due to the number of context-sensitive and opaque mappings (Fernandes et al., 2008; Morais, 1997), children with stronger orthographic knowledge may be better able to risk using less frequent or orthographically complex words in their writing, thereby increasing lexical diversity.

In this sense, the present results complement earlier research in European Portuguese that highlighted the role of foundational skills, such as complex grapheme-sounding, in reading fluency (e.g., Fernandes et al., 2024). Together, these findings suggest that, in an orthography of intermediate depth, both multi-letter grapheme knowledge and lexical orthographic knowledge form a critical foundation for higher-order literacy outcomes: reading fluency, reading comprehension, and as shown here, the lexical richness of children’s written composition.

The present findings further suggest that lexical diversity may function as a sensitive indicator of children’s orthographic and linguistic development, capturing not only transcriptional accuracy but also children’s willingness and ability to recruit less frequent or morphologically complex vocabulary. This interpretation is consistent with evidence that orthographic knowledge supports the deployment of advanced lexical items and syntactically richer constructions (Johansson, 2008; Kim, 2020; Limpo & Alves, 2013), highlighting the need for multidimensional approaches to writing assessment in early schooling.

### 4.4. Implications for Models of Writing

The present findings have implications for developmental models of writing, particularly the Simple and Not-So-Simple Views of Writing (Berninger et al., 1998; Berninger & Winn, 2006; Juel et al., 1986; Juel, 1988). These frameworks typically conceptualize spelling as a core component of transcription, which in turn supports higher-level composing processes. These models emphasize that transcription skills, including handwriting and spelling, are foundational for writing development, and that individual differences in transcription constrain text generation, particularly in the early grades. Our results suggest that the foundational skills underlying spelling, especially lexical orthographic knowledge, may themselves warrant explicit consideration in models of writing.

Although spelling is traditionally treated as a unitary construct in such models, the distinction between lexical and sublexical orthographic knowledge appears to be meaningful for understanding early writing development. In our data, once these components were included in the model, word spelling itself did not show a significant unique contribution to written composition in the more complex models. This does not imply that spelling is unimportant; instead, it suggests that, in the present design and at this developmental stage, the more proximal explanatory power resides in the orthographic knowledge that supports spelling. For semi-transparent orthographies like European Portuguese, where spelling demands sustained attention to complex and context-dependent grapheme–phoneme mappings (Fernandes et al., 2008; Sucena et al., 2009), foundational orthographic knowledge may be particularly critical for enabling efficient writing.

These findings therefore support an extension of current models of writing to include explicit orthographic components within the broader category of transcription skills, alongside handwriting and spelling. They are also consonant with broader literacy frameworks in which low-level processing (e.g., grapheme-sound knowledge, decoding, orthographic processing) continues to shape higher-level outcomes such as text fluency or comprehension across development (Fernandes et al., 2017, 2024; Vaessen et al., 2010).

Importantly, our findings complement recent proposals advocating for more explicit integration of orthographic processing within hierarchical models of writing (e.g., Kim, 2020). These frameworks argue that orthographic knowledge is not merely a precursor to spelling but a multilevel linguistic resource that influences lexical selection, morphological generation, and syntactic formulation. The current evidence adds empirical support to this view, demonstrating that orthographic processing predicts children’s writing outcomes even after controlling for spelling, and thus suggesting that foundational orthographic mechanisms perform functions that extend beyond transcriptional accuracy.

### 4.5. Limitations and Directions for Future Research

Several limitations of the present study should be acknowledged. First, the sample size, although adequate for the estimated models, restricted the complexity of the structural analyses that could be conducted. With 80 children with complete data, it was not feasible to test more elaborate models including additional predictors or moderators (e.g., gender, socio-economic status, oral language measures, handwriting fluency, morphological awareness, or executive functions), nor to perform multi-group comparisons. It is possible that including such variables would attenuate or qualify some of the effects observed here, for example, by revealing differential pathways for boys and girls or for children with different profiles of oral language or motor skills. For example, recent studies have shown that morphological awareness, vocabulary breadth, and handwriting fluency exert significant influence on children’s written composition even at early stages (e.g., Kim, 2020). Without including these constructs, our ability to determine the unique versus shared variance accounted for by orthographic knowledge is necessarily constrained.

Second, the assessment of written composition was limited to a single indicator, i.e., lexical diversity based on the number of different correctly spelled words, derived from a timed, single-occasion writing task on a familiar topic. Although lexical diversity is a well-established proxy for writing quality, it does not capture other important dimensions of writing, such as macro-organization, coherence, syntactic complexity, or genre-specific features (McNamara et al., 2010; Olinghouse & Wilson, 2013). Future studies should complement lexical indices with holistic or analytic ratings of writing quality to examine whether the patterns observed here generalize to broader conceptions of writing performance.

Third, the present study focused on a single school year (Grade 2) and on a narrow developmental window. Although this focus was theoretically motivated (Grade 2 being a period in which transcription processes remain highly demanding) it limits inferences about how the relations among orthographic knowledge, spelling, and writing evolve at later stages, when higher-level processes and discourse-level skills are likely to assume a more prominent role. Longitudinal work spanning several grades would be valuable to determine whether the strong direct contribution of lexical orthographic knowledge observed here is sustained, diminishes, or is superseded by other predictors as writing develops.

Finally, the sample consisted of children from two schools in the Lisbon district, predominantly from middle to upper-middle socio-economic backgrounds, and all instruction followed a phonics-based approach. As a result, the generalizability of the findings to other regions, socio-economic contexts, or instructional approaches is necessarily limited. Replicating this work in more diverse samples and in other orthographies of varying depth would help clarify the extent to which the observed patterns reflect universal processes or language-specific characteristics.

Future work should also consider longitudinal designs that incorporate intermediate assessments, allowing researchers to test growth trajectories in orthographic knowledge and writing and to examine whether the predictive power of lexical orthographic knowledge strengthens, plateaus, or becomes superseded by discourse-level processes across grades.

## 5. Conclusions

The present study investigated how lexical and sublexical orthographic knowledge measured at the beginning of Grade 2 relates to written composition at the end of the same school year in European Portuguese. The findings show that orthographic knowledge, particularly its lexical component, plays an important role in children’s writing, accounting for meaningful variance in both word spelling and lexical diversity in written texts. Lexical orthographic knowledge emerged as a strong direct predictor of written composition, whereas sublexical orthographic knowledge showed a weaker, fully mediated contribution through spelling.

These results extend previous evidence on the role of orthographic knowledge in reading and spelling to the domain of writing, highlighting that foundational orthographic processes contribute to writing development beyond their role in transcription accuracy. In an orthography of intermediate depth such as European Portuguese, where spelling is relatively more demanding than reading, both lexical and sublexical orthographic knowledge appear to support early writing, with lexical knowledge assuming a particularly prominent role.

By demonstrating that orthographic knowledge contributes not only to transcription accuracy but also to the lexical richness of children’s writing, the present study underscores the need to reconceptualize transcription as a multidimensional construct. Such reconceptualization may have important instructional implications, suggesting that explicit teaching targeting the development of lexical orthographic representations, through repeated exposure, morphological analysis, and graphotactic pattern instruction, may foster more expressive and lexically diverse writing in the early grades (González, 2017; Pacton & Commissaire, 2024).

More broadly, the present findings suggest that models of writing development should consider not only spelling and handwriting as core transcription skills, but also the underlying orthographic knowledge that sustains these skills. Understanding how these foundational processes interact with higher-level linguistic and cognitive abilities across development remains an important goal for future research in writing and literacy.

## Figures and Tables

**Table 1:** Means, Standard Deviations and Raw-Score Ranges (Min–Max) for Accuracy Measures and Written Composition at the Beginning and End of Grade 2.

	Grade 2					
	Beginning			End		
	M	SD	Range	M	SD	Range
Orthographic Choice Task (accuracy)	0.72	0.16	0.38–0.97	-	-	-
Corrected values	0.64	0.20		-	-	-
Orthographic Awareness Task (accuracy)	0.76	0.16	0.43–1.00	-	-	-
Corrected values	0.52	0.30		-	-	-
Word Spelling Task	0.64	0.13	0.31–0.97	-	-	-
Written Composition Task	-	-	-	-	-	-
Number of different words	-	-	-	65.89	23.45	24–119

*Note. N* = 80. Chance-corrected accuracy reflects correction for guessing (0.20 for the Orthographic Choice Task; 0.50 for the Orthographic Awareness Task). Values represent means and standard deviations after removal of outliers.

**Table 2:** Correlation Matrix for All Measures.

	Variable	1	2	3	4
1	Orthographic Choice Task at the beginning of Grade 2	1	0.27 *	0.63 **	0.38 **
2	Orthographic Awareness Task at the beginning of Grade 2		1	0.38 **	0.23 *
3	Word Spelling Task at the beginning of Grade 2			1	0.34 **
4	Written Composition Task at the end of Grade 2				1

*Note*. *N* = 80. * *p* < 0.05. ** *p* < 0.01.

**Table 3:** Model fit indices.

Model	*Χ* ^2^	*df*	*p*	CFI	RMSEA (a)
1—Direct	0.00	0	saturated model		
2—Total mediation	5.34	2	0.07	0.95	0.15 [0.00; 0.30]
3—Partial mediation	0.00	0	saturated model		
3.1—Partial mediation	2.61	2	0.27	0.99	0.06 [0.00; 0.24]

*Note.* (a) Confidence intervals at 90% for RMSEA; *p*-value refers to the test H0: RMSEA ≤ 0.05. Values of *p* > 0.05 indicate good fit.

**Table 4:** Squared multiple correlations (*R*^2^) of endogenous variables for each grade/model.

Model	Variables	*R* ^2^
1	Writing Composition	0.17 *
2	Word Spelling	0.44 **
	Writing Composition	0.12 *
3	Word Spelling	0.44 **
	Writing Composition	0.18 **
3.1	Word Spelling	0.44 **
	Writing Composition	0.15 *

*Note.* * *p* < 0.01; ** *p* < 0.05.

**Table 5:** Effects of Orthographic Knowledge on Writing Composition.

Lexical		St. Est.	90%CI for St. Est.	*p*
Model 1—without mediation	Direct effect	0.35	[0.15; 0.52]	0.00
Model 2—full mediation	Indirect effect	0.20	[0.10; 0.30]	0.00
Model 3—partial mediation	Direct effect	0.27	[0.05; 0.49]	0.06
	Indirect effect	0.07	[−0.04; 0.19]	0.26
	Total effect	0.35	[0.15; 0.52]	0.00
Model 3.1—partial mediation	Direct effect	0.38	[0.20; 0.53]	0.00
Sublexical				
Model 1—without mediation	Direct effect	0.14	[−0.06; 0.31]	0.27
Model 2—full mediation	Indirect effect	0.08	[0.02; 0.15]	0.01
Model 3—partial mediation	Direct effect	0.11	[−0.09; 0.28]	0.37
	Indirect effect	0.03	[−0.01; 0.09]	0.24
	Total effect	0.14	[−0.06; 0.31]	0.27
Model 3.1—partial mediation	Not Tested			

*Note. p*-values are reported for all effects.

## Data Availability

The data are not publicly available due to ethical and privacy restrictions related to participant confidentiality but are available from the corresponding author upon reasonable request.
